# Characterization of the Microflow Through 3D Synthetic Niche Microenvironments Hosted in a Millifluidic Bioreactor

**DOI:** 10.3389/fbioe.2021.799594

**Published:** 2021-12-17

**Authors:** Bogdan Ene-Iordache, Chiara Emma Campiglio, Manuela Teresa Raimondi, Andrea Remuzzi

**Affiliations:** ^1^ Department of Biomedical Engineering, Istituto di Ricerche Farmacologiche Mario Negri IRCCS, Ranica, Italy; ^2^ Department of Management, Information and Production Engineering, University of Bergamo, Dalmine, Italy; ^3^ Department of Chemistry, Materials and Chemical Engineering “Giulio Natta”, Politecnico di Milano, Milan, Italy

**Keywords:** miniaturized optically accessible bioreactor, NICHOID, stem cells, computational fluid dynamics, microflow

## Abstract

**Background:** Development of new medicines is a lengthy process with high risk of failure since drug efficacy measured *in vitro* is difficult to confirm *in vivo*. Intended to add a new tool aiding drug discovery, the MOAB-NICHOID device was developed: a miniaturized optically accessible bioreactor (MOAB) housing the 3D engineered scaffold NICHOID. The aim of our study was to characterize the microflow through the 3D nichoid microenvironment hosted in the MOAB-NICHOID device.

**Methods:** We used computational fluid dynamics (CFD) simulations to compute the flow field inside a very fine grid resembling the scaffold microenvironment.

**Results:** The microflow inside the multi-array of nichoid blocks is fed and locally influenced by the mainstream flow developed in the perfusion chamber of the device. Here we have revealed a low velocity, complex flow field with secondary, backward, or local recirculation micro-flows induced by the intricate architecture of the nichoid scaffold.

**Conclusion:** Knowledge of the microenvironment inside the 3D nichoids allows planning of cell experiments, to regulate the transport of cells towards the scaffold substrate during seeding or the spatial delivery of nutrients and oxygen which affects cell growth and viability.

## Introduction

The development of a new drug is subjected to regulatory approvals following three stages: laboratory discovery *in vitro*, animal testing *in vivo* and clinical trials on patients. This process may last longer than 10 years but, among thousands of new molecules, only one arrives to the market. Thus, drug development is a process with very high failure rate in the pre-clinical testing phase since drug efficacy measured *in vitro* is almost never confirmed in animals (Scannell et al., 2012). In fact, many pharmaceutical companies do not develop new drugs because costs exceed 1 billion euros (Ashburn and Thor, 2004), (Pammolli et al., 2011).

With the aim to add new device in drug discovery for testing the effects of drugs on 3D tissue-equivalents and organoids, a miniaturized optically accessible bioreactor (MOAB) was developed (Raimondi et al., 2020). Thanks to the optical transparency and low thickness of the components, it is optically accessible and may be exploited as a 4D bioreactor for prolonged culture. The MOAB may be used to assess the effect of several parameters on engineered tissue growth with time by viable staining and standard fluorescence microscopy. Moreover, the MOAB can be used as stand-alone or can be combined with several types of cell culture substrates, like for example bone matrices (Marturano-Kruik et al., 2018), hydrogel substrates (Tunesi et al., 2016), or the microscopic NICHOID scaffold.

The “nichoid” is a novel cell substrate inspired by the natural stem cells’ niche. This polymer-engineered scaffold is composed of three-dimensional (3D) grids composed of horizontal and vertical rods that perfectly recreate the natural stem cell niche, ensuring at the same time good optical accessibility. Early studies on nichoids as cell scaffold have obtained promising results. When cells were grown inside the nichoid they had a round nucleus, similarly to stem cells’ physiological morphology (Boeri et al., 2019). The nichoids promoted pluripotency of embryonic stem cells during expansion, counteracting cell migration between adjacent niche by means of its microarchitecture (Nava et al., 2016). Rat mesenchymal stem cells seeded and grown in the nichoids fabricated directly onto circular glass cover slips, had reduced cell motility and nuclear pore dimensions and increased expression of the mechano-transducer transcription factor YAP (Remuzzi et al., 2020). These mechanobiology effects may be useful for preserving cell stemness and function during *in vitro* culture to potentially obtain more favorable effects in cell therapy.

Combining the nichoids into the MOAB allows to culture 3D organoids of few millimeters in size under continuous perfusion of the culture medium, infusion of drugs to be tested and diagnose of the cell response either in real time or post-cultivation. The flow microenvironment inside the 3D nichoids modulates the transport of cells towards the scaffold substrate during cell seeding or the spatial distribution of nutrients and oxygen which is related to cell growth and viability (Olivares and Lacroix, 2012; Nava et al., 2013; Hossain et al., 2015). Moreover, the fluid shear stress exerted on cells affects cell response (Sikavitsas et al., 2003; Becquart et al., 2016). Therefore, acquisition of the spatial fluid flow conditions inside the 3D scaffold is essential to understand the fluid-induced cell behavior, the convective transport of solute and to control tissue development. The aim of our study was thus to characterize the microflow through the 3D nichoid microenvironment hosted in the MOAB-NICHOID device.

## Materials and Methods

### The MOAB-NICHOID Device

**FIGURE 1:**
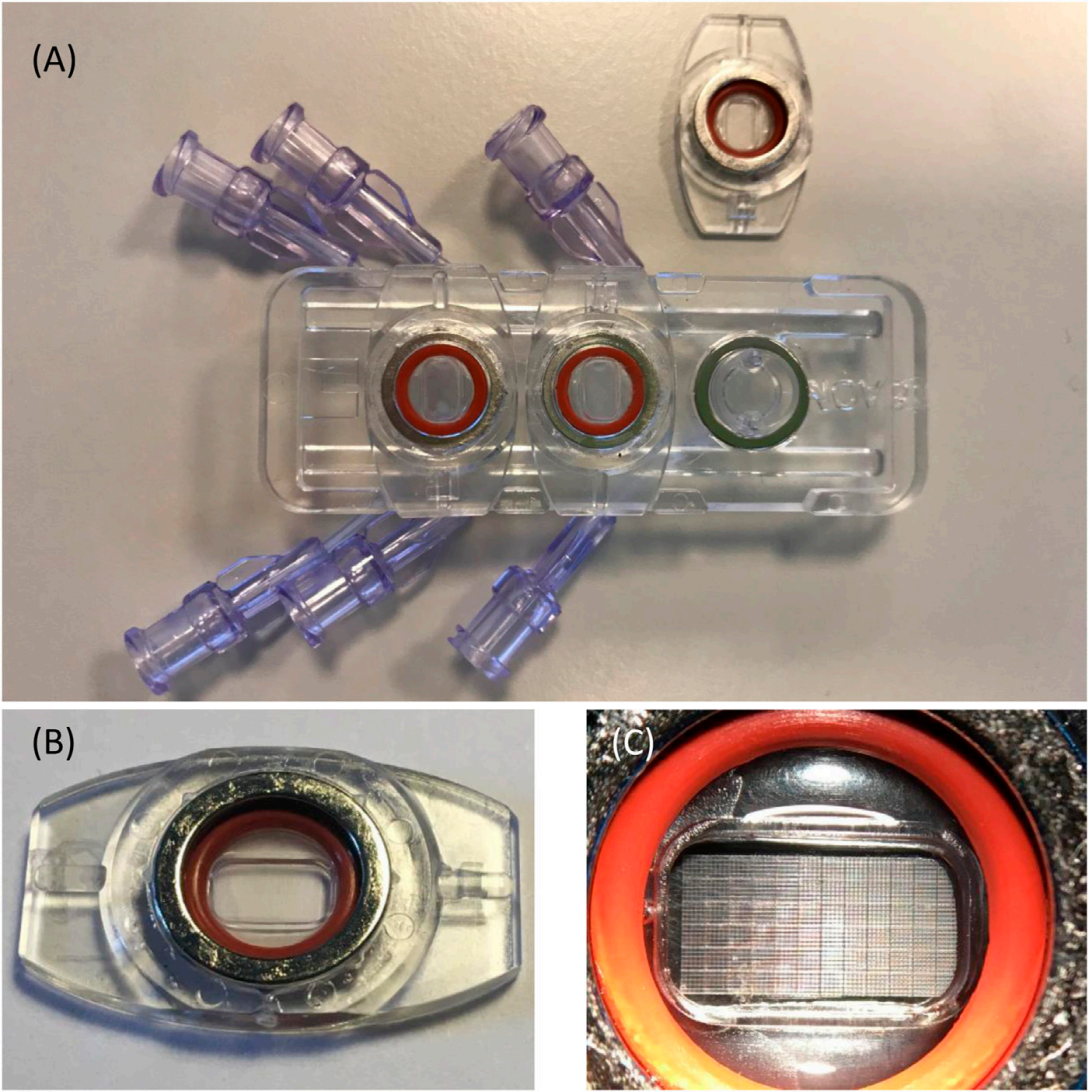
The MOAB-NICHOID bioreactor. **(A)** The device is composed of three perfusion chambers, one is open; **(B)** The removable part containing the perfusion chamber; **(C)** Zoom on **(B)** showing the nichoid blocks on the bottom of perfusion chamber.

The MOAB-NICHOID is basically the integration of the nano-engineered 3D substrate for stem cell culture nichoid into the MOAB, as shown in Figure 1. The MOAB-NICHOID device has three separate perfusion lines with optically accessible micro-chambers (Figure 1A) which allow to simultaneously cultivate three independent scaffolds in the same experiment. Each micro-chamber is composed of two parts, one fixed and one removable that can be easily sealed through a magnet with O-ring system (Figures 1A,B). The removable part contains the perfusion chamber itself with blocks of nichoids on its bottom (Figure 1C) where the cells are cultured. Since the removable part can be inspected by common microscopes, the MOAB-NICHOID device allows culturing in triplicate 3D tissue equivalents of few million cells, that can be further harvested and analyzed during and at the end of experiments.

#### The Perfusion Chamber of MOAB-NICHOID Device

**FIGURE 2:**
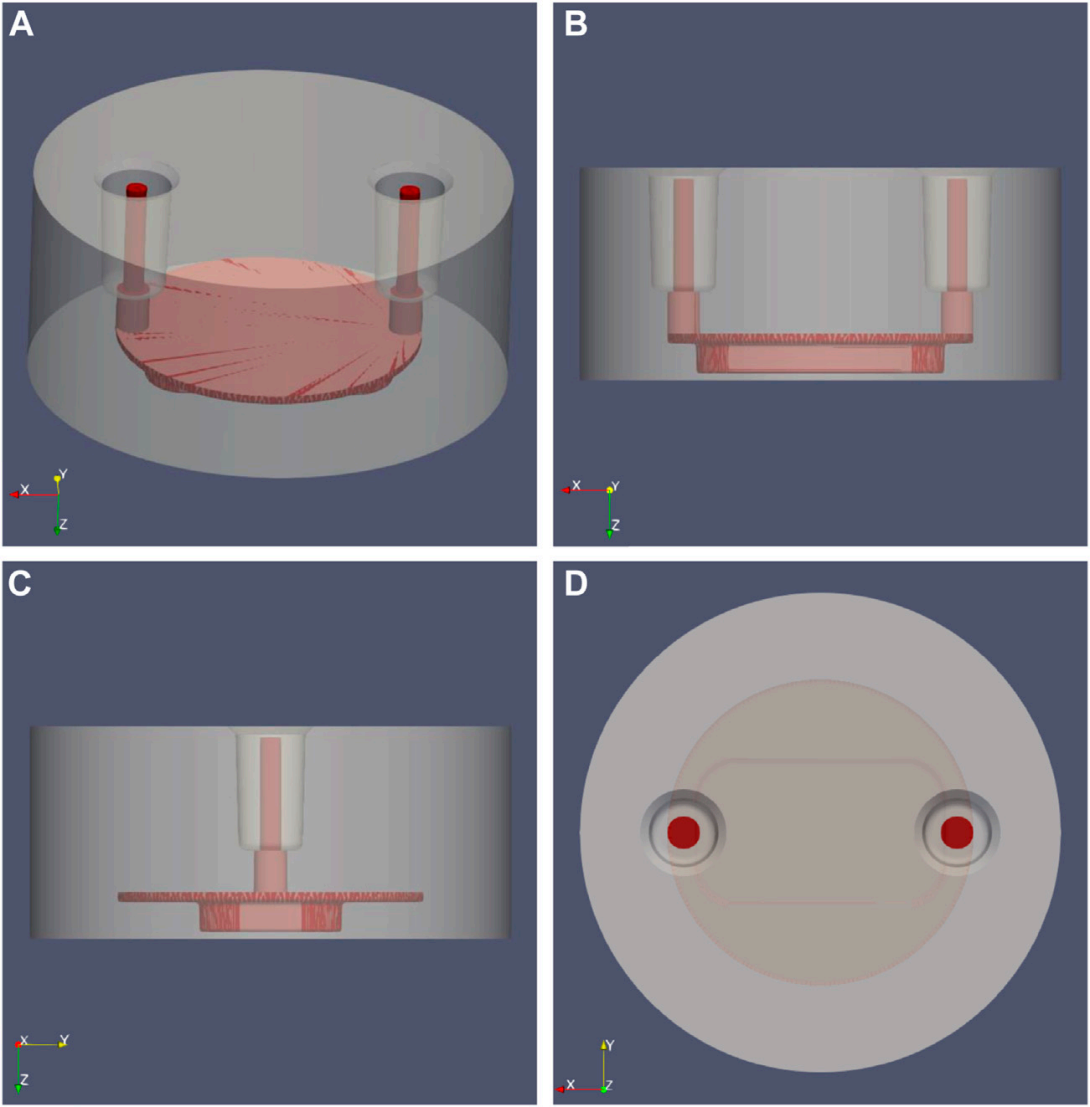
MOAB-NICHOID perfusion chamber: the main body (grey) was set transparent to reveal the shape of the internal fluidic path (red). **(A)** Isometric view; **(B)** Side view from Y axis; **(C)** Side view from X axis; **(D)** Top view, Z axis.

The perfusion chamber of the MOAB device is formed by a body and a lid as shown in Figure 2. The cylindrical body (diameter 11.6 mm x height 5.15 mm) is made of plexiglass in which the fluidic path is excavated. The fluidic path is made of two vertical cylinders for flow inlet and outlet, made by microbore tubes of internal diameter 0.5 mm, which are connected to the scaffold chamber as shown in Figure 2. On the bottom of the scaffold chamber there is a lid made of round cover glass for microscopy, 0.16 mm in thickness, on which nichoids can be seeded in multiple blocks.

#### The 3D NICHOID Scaffold

**FIGURE 3:**
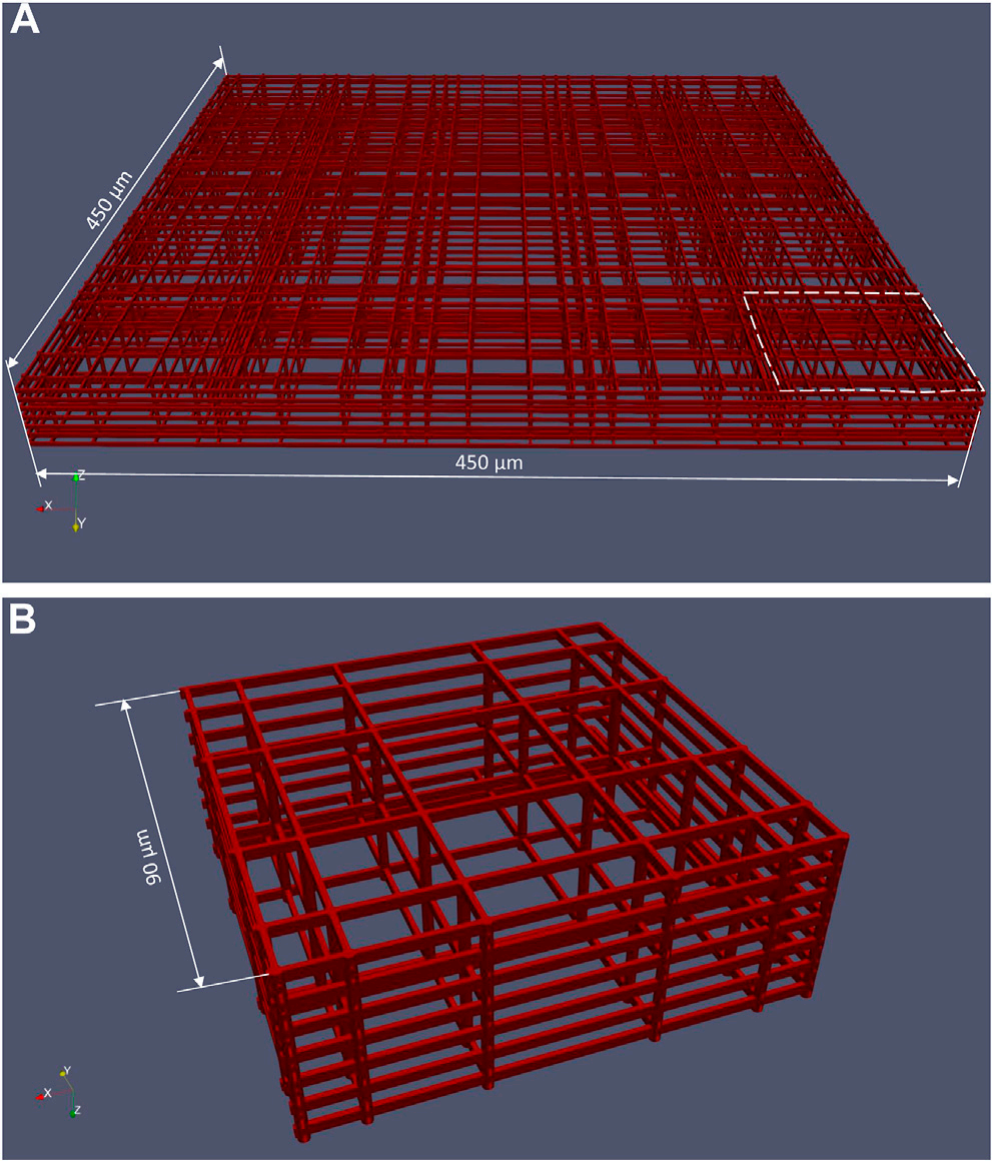
CAD file of a basic nichoid block. **(A)** A nichoid block is formed by 5 × 5 elementary niche; **(B)** Elementary niche (shown in the dashed area in **(A)**).

The structure of the 3D cell culture support is composed by a matrix of 12 × 6 basic blocks (Figure 1C) manufactured on the bottom of the perfusion chamber. The spacing between adjacent basic blocks is 30 μm. As shown in Figure 3A, one basic block is made of interconnected horizontal and vertical rods of 2 μm in size, the whole block being 33 μm high and 450 μm wide. The basic block itself is composed of 5 × 5 elementary niches that are 90 μm wide (Figure 3B). Of note, the external walls of an elementary niche are formed by 7 horizontal rods, while the intermediate walls are formed by 3 horizontal rods only. Nichoid blocks are polymerized with a laser in biocompatible resin (SZ2080 photoresist) using “two-photon laser polymerization” as described in detail previously (Ricci et al., 2017).

#### Dimensional Assessment of the Nichoids Seeded on the MOAB-NICHOID Device

To obtain the real dimensions of the nichoid blocks inside the MOAB perfusion chamber we analyzed the nichoids on images acquired at scanning electron microscopy (SEM). Five measurements of basic nichoid blocks and of the distance between them were acquired and averaged as shown in Table 1. According to these data, the side length of a basic block that should be theoretically 450 μm is in reality about 434 μm, while the distance between two blocks that should be theoretically 30 μm, is in reality around 20 μm in average. Thus, we have found that there is a physical contraction of individual blocks in the process of fabrication of the nichoid substrate of the bioreactor.

**TABLE 1:** Measurements of basic (5 × 5 niche) nichoid blocks and of the distance between blocks.

#	5 × 5 block side (μm)	Distance between blocks (μm)
1	434.786	19.135
2	428.873	19.462
3	433.642	20.229
4	436.529	18.895
5	436.529	18.911
average	433.677	19.326
SD	2.88	0.55

### Validation of Pressure Losses in the MOAB Flow System

#### Experimental Measurements of Pressure Inside the MOAB Circuit

**FIGURE 4:**
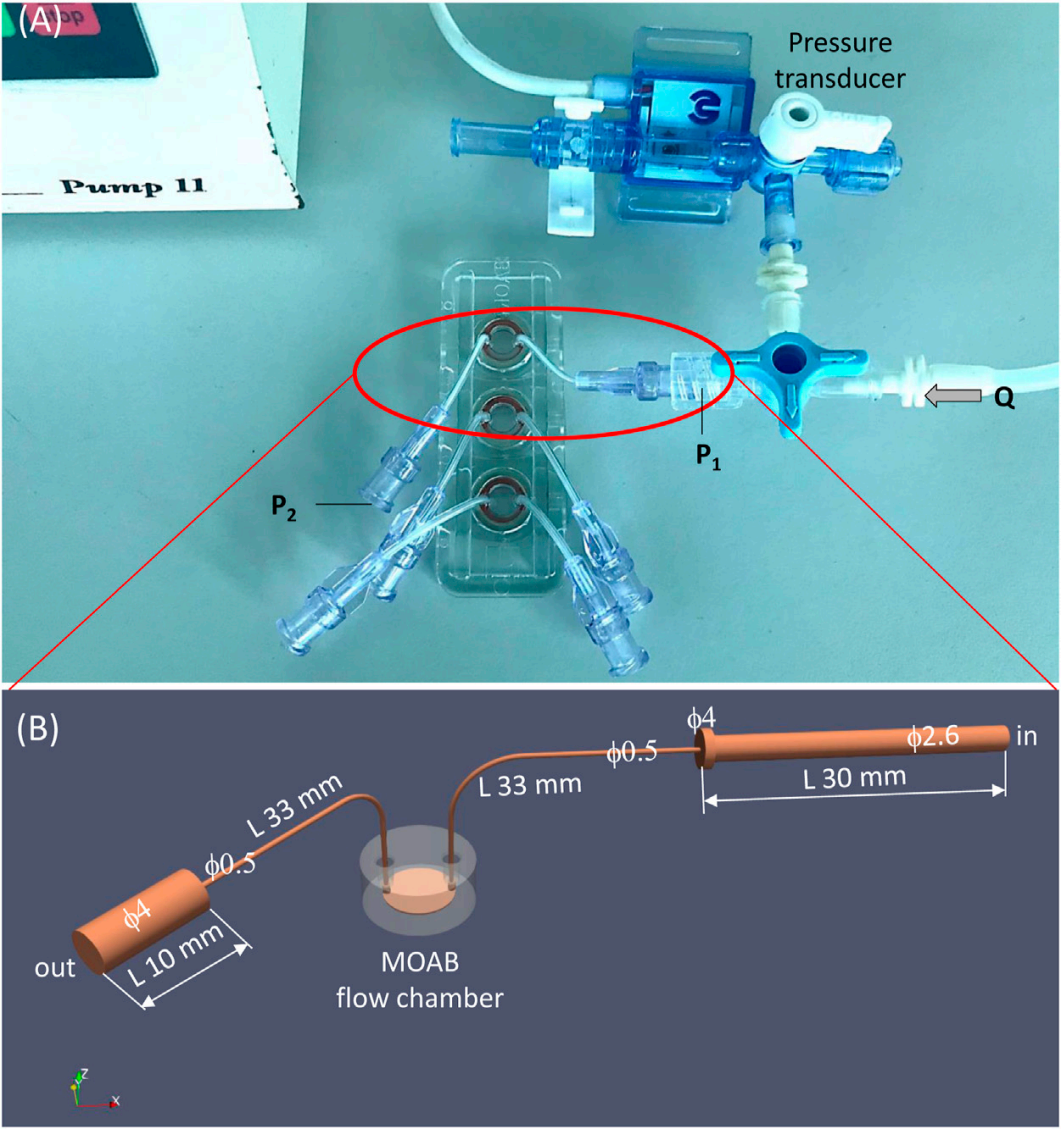
The experimental setting for measurement and validation of pressure drop in the MOAB flow system. **(A)** Experimental setting composed of syringe pump, silicone rubber tubing circuit, pressure transducer and the MOAB flow system; **(B)** CAD model of the MOAB circuit shown in **(A)**: inlet luer and tube, scaffold chamber and outlet tube with luer.

We measured experimentally the pressure drop over the MOAB flow system. The experimental flow system is shown in Figure 4A. A syringe pump (Pump 11, Harvard Apparatus, Holliston, MA) circulates water at 24°C in a silicone rubber tubing circuit in which a pressure transducer (MP150 Biopac Systems, Goleta, CA) and the MOAB device are included. Using a flow rate of 2.295 ml/min, the experimentally measured pressure drop was 11.86 mmHg.

#### CFD Simulation of the MOAB Flow Circuit

Full details of the numerical simulation of the MOAB full circuit are provided in the Supplementary Material. Briefly, a detailed model of the MOAB flow circuit was generated using a CAD program (FreeCAD, https://www.freecadweb.org/). The entire fluid path surface was exported in stereolithography (STL) file format and then was sub-divided into separate parts representing the patches used in CFD simulations like inlet, outlet, and walls. The computational grid of the MOAB flow circuit was generated with the *snappyHexMesh* pre-processor of the OpenFOAM CFD toolbox (OpenCFDLtd, 2020). A fine mesh composed of more than 5.7 million cells was generated. Steady simulations were run using the solver *simpleFoam* of OpenFOAM suite. The fluid considered was the same used in experimental measurements of pressure, i.e., water at 24°C, for which we assumed density (ρ) equal to 0.9973 g cm^−3^ and dynamic viscosity (µ) equal to 0.0091 g cm^−1^·s^−1^. Newtonian rheology model was assumed. As boundary conditions, we set a constant volumetric flow rate at the inlet equal to that used in the experimental setting, which was 2.295 ml/min. On the outlet, a zero-pressure condition was set, and no-slip condition was set on all system walls.

To derive the characteristic flow-pressure curve of the whole circuit, as well as of the scaffold chamber alone, we have run steady simulations with a set of volumetric flow rates and medium commonly used *in vitro* experiments on cells using the MOAB device. For the medium for cells at 37°C we assumed a density of 0.99 g cm^−3^ and a dynamic viscosity of 0.0076 g cm^−1^·s^−1^ (Franzoni et al., 2016).

### Generation of the Computational Grid of the Flow Chamber Including the Scaffold

Since we are mainly interested in the flow field inside the 3D scaffold, a fine computational mesh must be generated in the region of perfusion chamber with nichoids seeded on its bottom. Of note, the geometric scale has different orders of magnitude e.g., the fluidic path of the chamber is in mm (order 10^–3^ m), whereas the nichoid structures are composed of multiple rods having thickness of 2 μm (order 10^–6^ m). Therefore, we have performed a preliminary meshing study of a basic nichoid block and of the fluidic path chamber (see Supplementary Material). Regarding the grid of a nichoid block, we have found that it must be refined at the μm level, otherwise its rods, especially the horizontal ones, will not be created and distorted cells will be generated nearby. According to this analysis, a nichoid block should have more than 5.4 million cells to achieve a well refined mesh for accurate numerical solution. Since the real substrate of nichoids is composed of a matrix of 12 × 6 blocks, for which the total mesh would result in more than 380 million cells, we decided to consider the symmetry of the chamber to reduce the computational grid.

**FIGURE 5:**
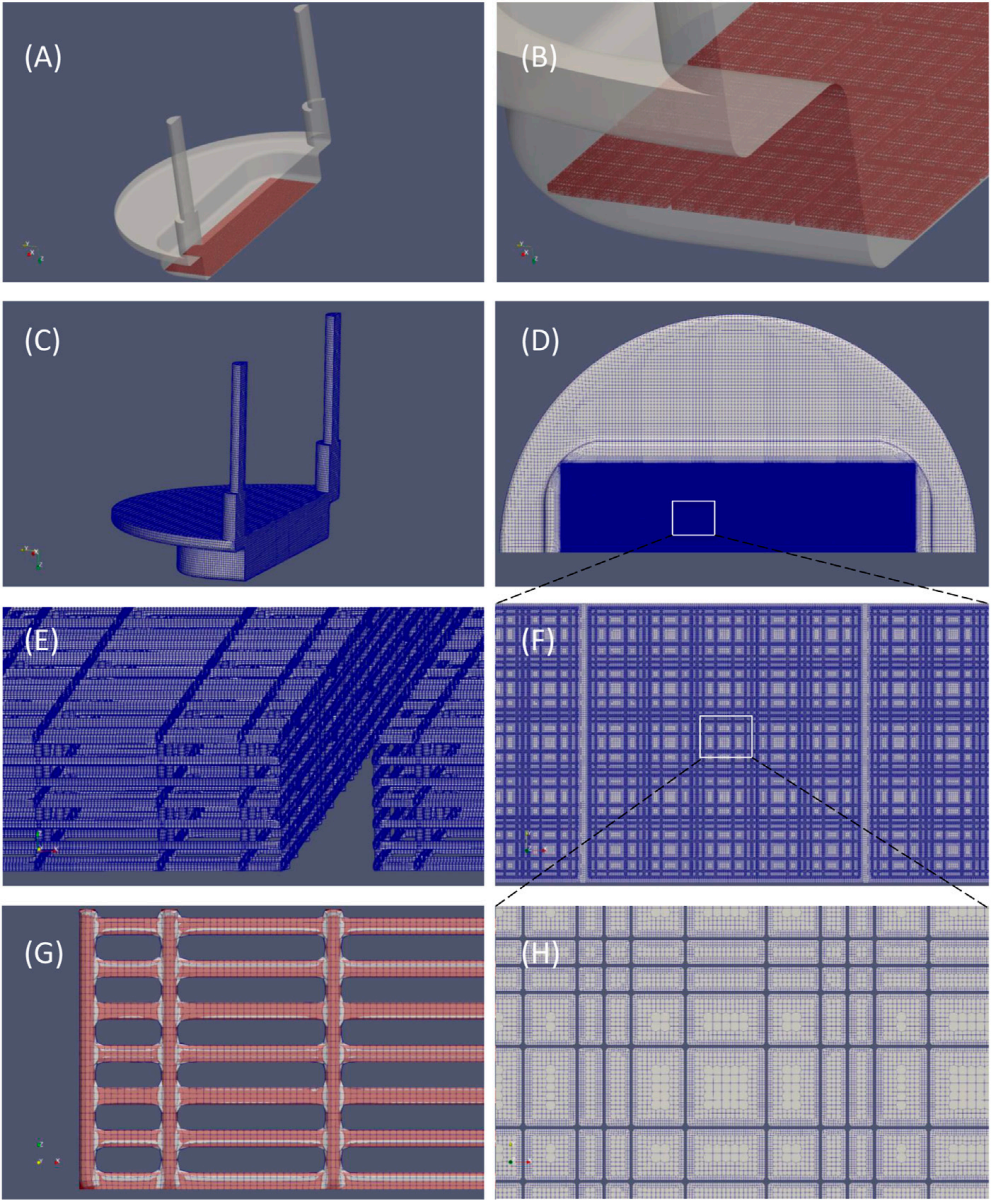
Original CAD files and the computational grid. **(A)** Isometric view of the fluidic path chamber (in transparent grey) and nichoid blocks arranged in a 12 × 3 matrix (in red); **(B)** Detail of **(A)** showing the three rows of nichoid blocks; **(C)** The computational mesh; **(D)** View of **(C)** seen from bottom, showing the nichoid scaffold zone; **(E)** Detail of the grid of nichoid rods; **(F)** Detail of the area in **(D)** showing the fine grid in the nichoid zone; **(G)** Comparison of the mesh (gray) with the original. stl file (in red); **(H)** Detail of the area in **(F)** showing the level of refinement in the nichoid zone.

The computational mesh was created starting from CAD files in stereolithography (.stl) format of both perfusion chamber and nichoid block. Three preparation steps were needed to setup the geometry for meshing. Firstly, the. stl file of a basic block (Figure 3) was re-scaled such as to match 434 μm width as found in the dimensional analysis described earlier. The second step was generation of a matrix of 12 × 3 nichoid blocks with a distance between blocks of 20 μm. Finally, as shown in Figures 5A,B, this matrix of blocks was rotated and translated such as to be aligned on the bottom of the fluidic-path chamber, as the real nichoid blocks in the MOAB-NICHOID bioreactor.

The computational grid was created using *cfMesh* generator of OpenFOAM CFD toolbox (OpenCFDLtd, 2020) by setting a very fine refinement level in the region of nichoid scaffold. The final mesh has more than 188 million of cells, the majority of which are hexahedral (Table 2). The mesh is shown in Figure 5. Obviously, the highest number of cells (>180 millions) are concentrated in the region of nichoid 3D scaffold where the refinement level reaches the μm resolution (see Figure 5D). Examples of nichoid rods refinement are shown in Figure 5E; Figure 5F. The numerical grid was carefully inspected visually and compared with the original surface (Figure 5G) to be sure that the entire shape of nichoid blocks is well preserved.

**TABLE 2:** Characteristics of the computational grid with 12 × 3 matrix of nichoid blocks.

Total N of cells	N of hexahedra	Faces/cell	N of non-orthogonal faces	N of skew faces
188,653,366	165,139,943 (88%)	6.3384	178	5

Moreover, the quality of the mesh was assessed with the *checkMesh* utility of OpenFOAM, as reported in Table 3. Regarding the quality checks, the computational grid is composed predominantly (88%) of hexahedral cells, and it has only 178 non-orthogonal and 5 skew faces, which is very low for such a huge mesh.

**TABLE 3:** Input volumetric flow rates and corresponding pressure drops.

Q inlet (*ml/min*)	Water at 24°C		Medium for cells at 37°C	
	ΔP_circuit_ (*mmHg*)	ΔP_flow chamber_ (*mmHg*)	ΔP_circuit_	ΔP_flow chamber_ (*mmHg*)
0.3	1.38	0.15	1.15	0.12
0.45	2.08	0.22	1.74	0.18
0.6	2.79	0.30	2.34	0.25
1.2	5.76	0.64	4.86	0.53
**2.295**	**11.67**	1.34	9.97	1.17
2.4	12.27	1.42	10.49	1.24

**Note**: in bold type values used in the experimental validation of numerical procedure.

### CFD Simulation of the Perfusion Chamber of MOAB-NICHOID Bioreactor

Steady flow simulations were run using the solver *simpleFoam* (OpenCFDLtd, 2020). A second order-accurate scheme was used for the discretization of convective terms. The residual control for the SIMPLE algorithm was set to 10^–4^ for both pressure and velocity. The fluid considered was medium for cells at 37°C for which Newtonian rheology was assumed. The physical characteristics of the medium were density (ρ) of 0.99 g cm^−3^ and dynamic viscosity (µ) of 0.0076 g cm^−1^·s^−1^ (Franzoni et al., 2016). As boundary conditions, we set a constant volumetric flow rate at the inlet equal to the flow rate of the infusion pump, which is 0.45 ml/min. Because we deal with symmetry which affect the flow entrance and exit as well (see Figure 5), the imposed flow rate was halved at the inlet i.e., 0.225 ml/min. On the vertical cutting wall, a boundary condition of symmetry was set. On the outlet, a zero-pressure condition was set, and no-slip boundary condition was set on all perfusion chamber and nichoids walls.

Due to the huge number of cells of the 3D domain in the scaffold zone, both grid generation as well as the numerical simulation were carried out on a High-Performance Computing (HPC) cloud infrastructure (https://cloudhpc.cloud/).

### Data Post-processing

**FIGURE 6:**
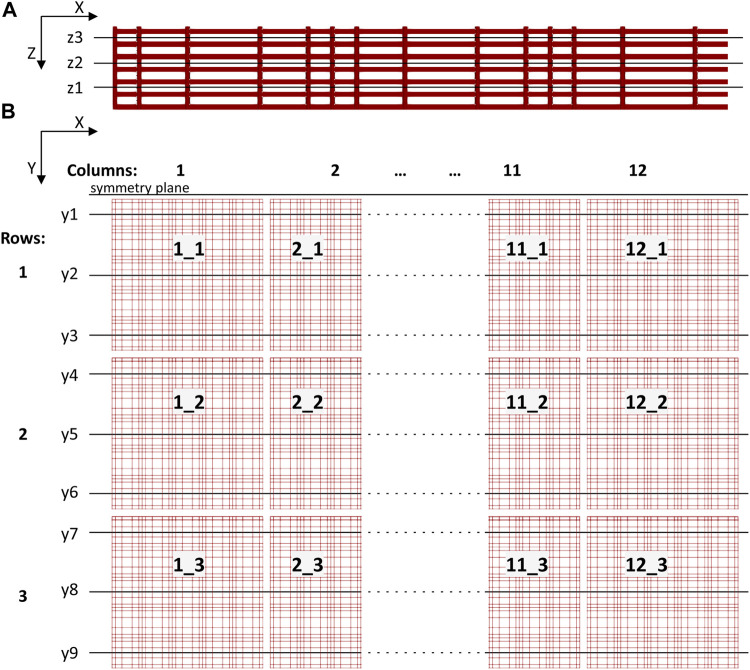
Sketch showing cutting planes for postprocessing through the 3D scaffold structure. **(A)** Three horizontal planes (z1 to z3, normal to Z axis) defined through a basic nichoid block (not in scale with **(B)**; **(B)** The 12 × 3 matrix of nichoid blocks seen from top and the vertical planes (y1 to y9, normal to Y axis).

Pressure and velocity fields from the 3D nichoid substrate were probed in vertical and horizontal planes cutting the 3D geometry. We identified three horizontal planes, normal to Z axis, as shown in Figure 6A. Precisely, plane z1 was defined at a height of 8.6 μm from the bottom of the perfusion chamber, plane z2 at a height of 18.6 μm, and z3 at a height of 28.6 μm. We also defined nine vertical planes (y1 to y9) normal to Y axis, traversing the nichoid blocks in the middle and more laterally, as shown in Figure 6B.

Moreover, we considered the 12 × 3 matrix organization of the cellular scaffold such as to name each block with an objective rule, considering the main direction of flow in the perfusion chamber, which is from left to right along the X axis. There are 12 basic nichoid blocks along the X axis and 3 blocks along the Y axis. A basic block is labelled with the X_Y rule i.e., first its position along the X axis and then its position along the Y axis.

Results of the numerical simulations such as general flow field, velocity and wall shear stress (WSS) patterns were post-processed with the open-source, data analysis and visualization program *ParaView* (Paraview, 2020).

## Results

### Pressure Losses in the MOAB Flow Circuit and in the Scaffold Flow Chamber

For the flow rate and type of fluid used in the experimental setting (e.g., 2.295 ml/min and water at 24°C), the numerical calculated pressure drop over the MOAB circuit resulted 11.67 mmHg. Since the experimentally measured pressure drop was 11.86 mmHg, we concluded that the numerical simulations may well predict the pressure losses over the MOAB flow system.

We therefore report in Table 3 the characteristic pressures vs. flow rate values of the entire MOAB flow system as well as of the scaffold chamber alone.

### General Flow Field of the Perfusion Chamber of MOAB-NICHOID Bioreactor

**FIGURE 7:**
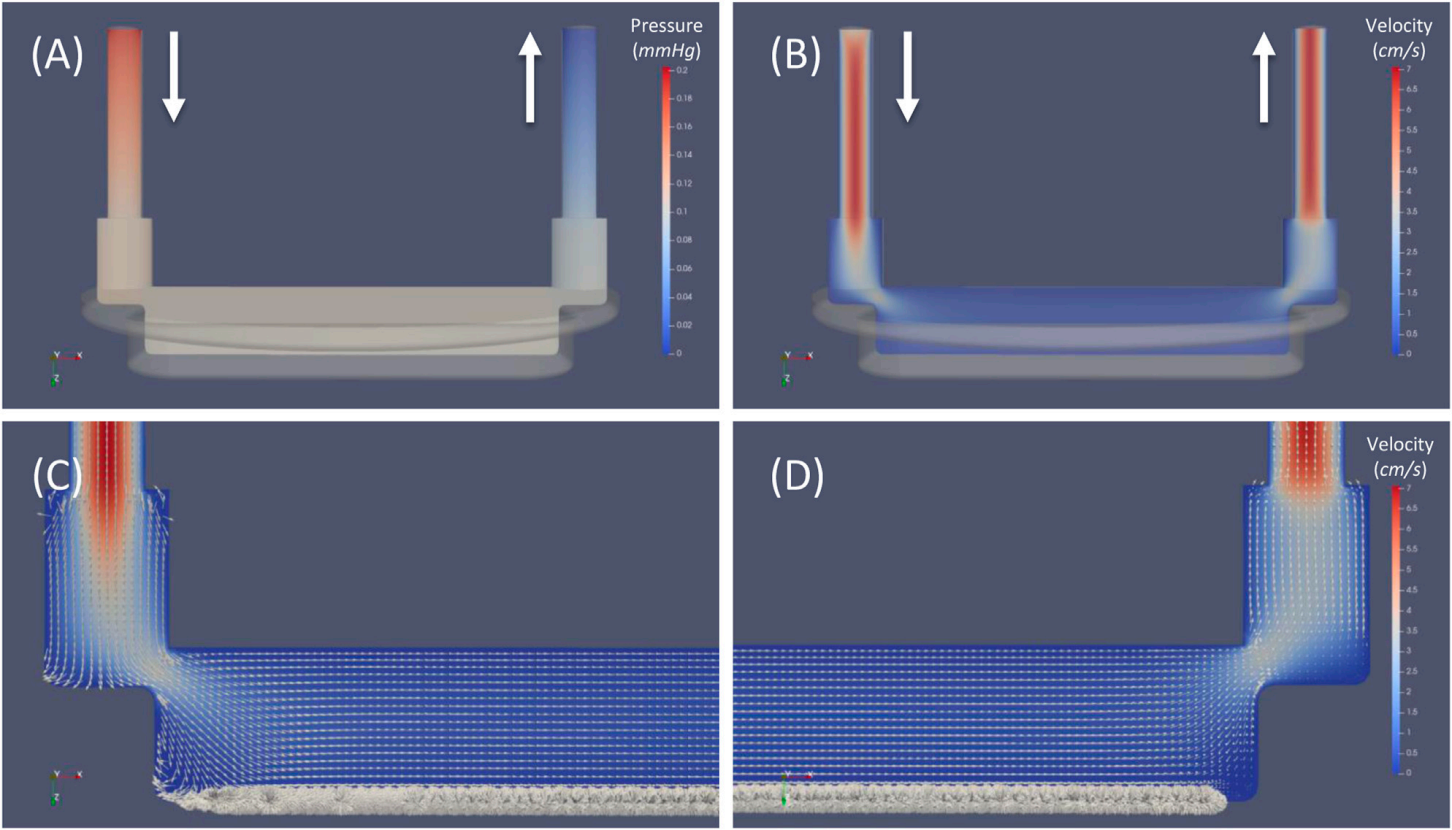
General flow field of the perfusion chamber of MOAB-NICHOID bioreactor. **(A)** Pressure contours; **(B)** Velocity magnitude contours; **(C)** Zoom view near the inlet zone; **(D)** Zoom view near the outlet zone. Note: superimposed vectors in **(C)** and **(D)** were normalized (set magnitude = 1); bold arrows indicate the inlet and outlet of the flow circuit.

The general flow field of the perfusion chamber of the MOAB-NICHOID bioreactor is shown in Figure 7. Pressure contours are shown in a plane very near the symmetry plane of the perfusion chamber (Figure 7A). The maximum pressure occurs at the chamber inlet, while zero pressure was set at the outlet. For this case we calculated a pressure drop of 0.19 mmHg over the entire flow chamber.

Velocity magnitude contours in the same plane are shown in Figure 7B. The maximum velocity around 7 cm/s is reached in both inlet and outlet vertical cylinders. As the fluid moves further from the inlet channel, the volume of perfusion chamber increases, and the velocity decreases proportionally.

To allow visualization of the flow features we first normalized all velocity vectors and then superimposed the normalized vectors over the velocity contours in Figures 7C,D. Such visualization allows to distinct well between the general flow of the chamber and the microflow inside the nichoid 3D substrate: while the flow in the chamber is smooth and laminar in the top layers, it becomes very complex and heterogeneous through the scaffold, owing to its complex 3D architecture. Although probed in a plane, most velocity vectors in the nichoid substrate region are oriented out of plane or even in opposite direction respect to the main direction of flow.

### Velocity Distribution Through the 3D Scaffold

**FIGURE 8:**
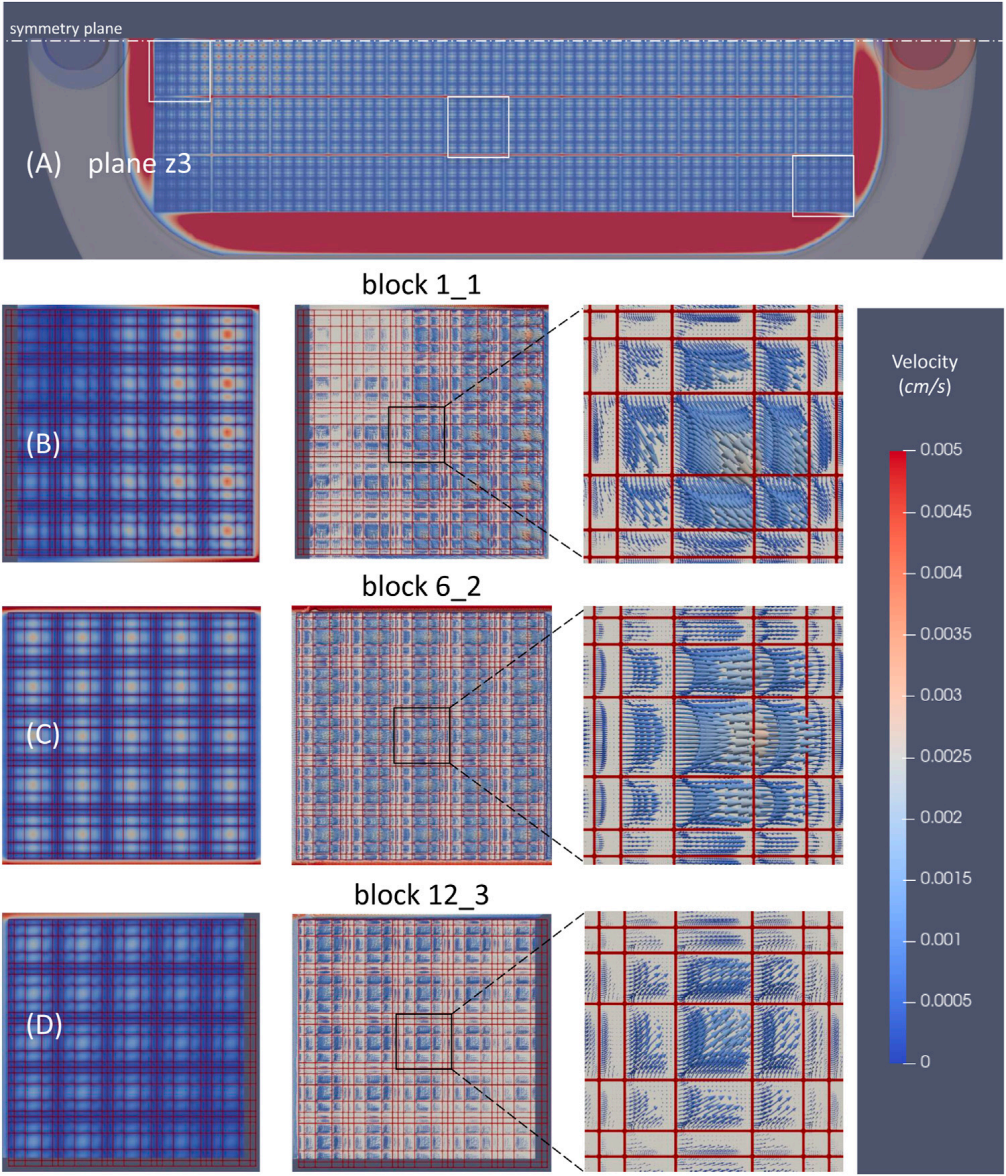
Velocity distribution through the 3D scaffold in the horizontal plane z3. **(A)** Velocity magnitude contours through all nichoid bocks; **(B)** Velocity magnitude contours and vectors in a block that is at the beginning of the matrix (1_1); **(C)** Velocity magnitude contours and vectors in a block in the middle (6_2); **(D)** Velocity magnitude contours and velocity vectors in the block at the end of the matrix (12_3). Note: the position of plane z3 is as shown in Figure 6A; velocity scale cutoff is 0.005 cm/s; the main direction of flow in the perfusion chamber is from left to right.

The flow velocities inside nichoid blocks resulted much lower than the corresponding velocities of upper free layers and turned out to be lower and lower towards the bottom of scaffold. We have calculated an average value of 5.6, 1.3, and 0.5, and a maximum value of 76, 8.8 and 2.1 μm/s for the three planes z3, z2 and z1, respectively. An example of the velocity field in the horizontal plane z3 is shown in Figure 8. It is worth mentioning that the maximum velocities are achieved in the longitudinal channels between adjacent nichoid blocks where the fluid is accelerated.

Velocity magnitude contours in all nichoid blocks revealed a rather non-uniform distribution in those blocks in vicinity of the entrance and exit of flow, and a uniform distribution in the central blocks, especially from the 4th up to the 9th columns (Figure 8A). The non-uniform distribution of velocity is better observed as vectors in the detailed view for the block 1_1, near the symmetry plane and near entrance, and for block 12_3, near the exit of the flow (Figures 8B, D). At the contrary, the velocity vectors inside the block 6_2 are all oriented in the same direction of the main flow (Figure 8C).

**FIGURE 9:**
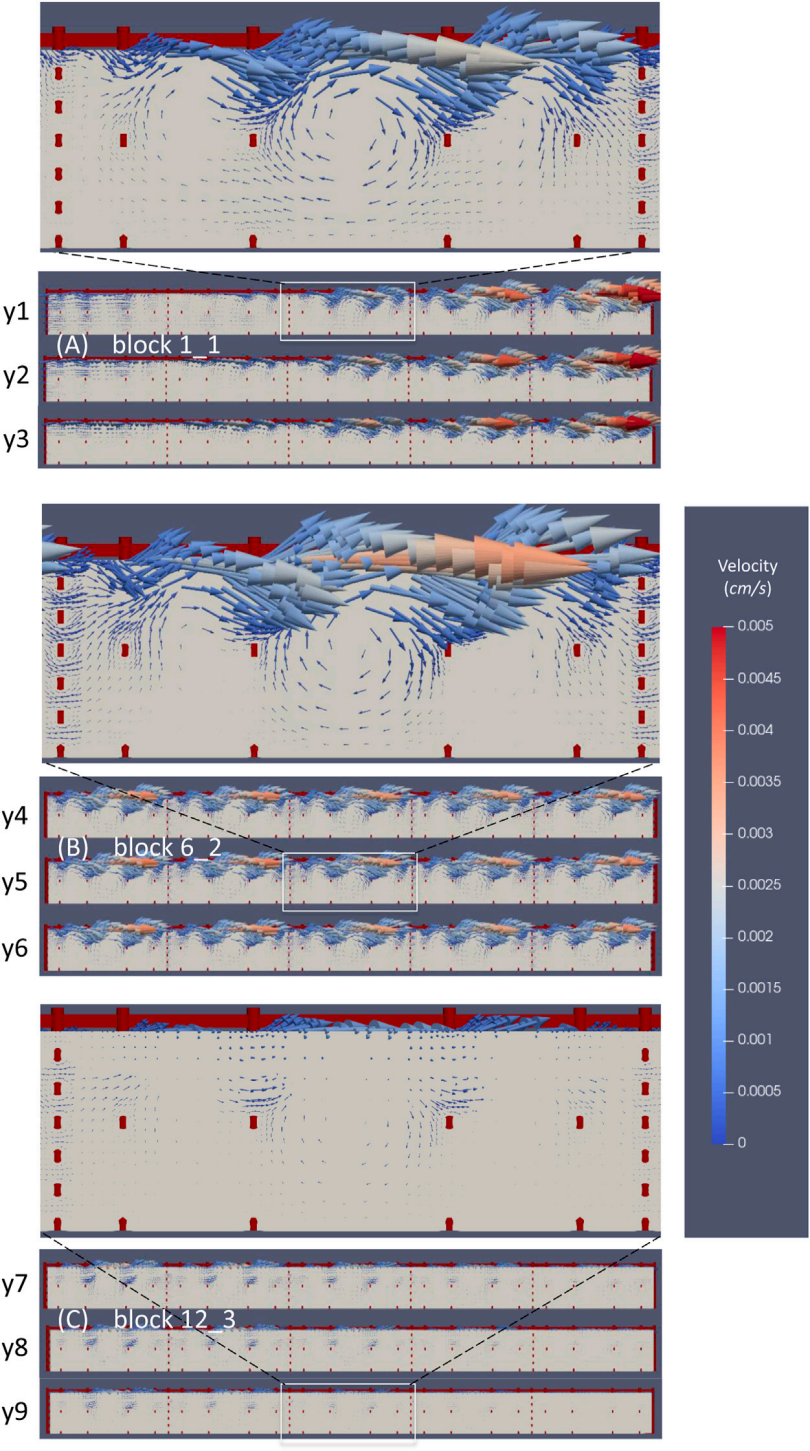
The microflow inside three nichoid blocks. **(A)** Velocity vectors probed in three vertical planes inside the nichoid block 1_1; **(B)** Velocity vectors probed in three vertical planes inside the nichoid block 6_2; **(C)** Velocity vectors probed in three vertical planes inside the nichoid block 12_3.

The velocity vectors probed in the vertical planes crossing the above three nichoid blocks are presented in Figure 9.

The flow inside the 3D scaffold is very complex, featuring recirculation zones and important secondary motions. Moreover, the flow inside the nichoid blocks is influenced locally by the main flow. For example, the big recirculation flow near the entrance affects the inner microflow that develops inside block 1_1. Figure 9A illustrates well how the velocity vectors in the left zone of the block are oriented opposite, whereas the vectors in the right region are oriented as the main flow. The detailed inset image reveals the microflows between the horizontal rods and recirculation flows developing in each single niche.

The velocity vectors at the upper part of block 6_2 are oriented in the same direction with the main flow of the perfusion chamber (Figure 9B). Also in this case, the inset image reveals microflows between the horizontal rods and recirculation flows.

The microcirculation inside block 12_3 is influenced by the main flow that here is oriented towards the outlet of the perfusion chamber (Figure 9C). In this case, the vectors are oriented diagonally out of plane and are better spotted in the inset image. Recirculation microflows are observed here, too.

### WSS Distribution on Scaffold Rods

**FIGURE 10:**
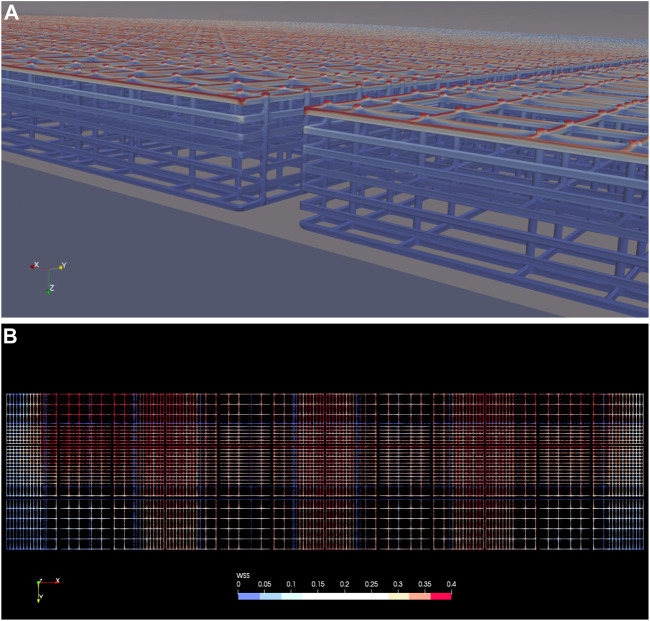
Wall shear stress on the nichoid rods. **(A)** WSS on the nichoid blocks, isometric view from the symmetry plane showing the space between the second and third columns of blocks; **(B)** WSS on all nichoid blocks seen from top. Note: a cutoff value of 0.4 dyne/cm^2^ was intentionally set as maximum.

Wall shear stress distribution on the nichoid blocks is presented in Figure 10. Due to the low velocities inside the scaffold blocks, the WSS is low on all rods except those on top that are hit directly by the mainstream fluid flow, as depicted in Figure 10A.

Patterns of WSS on the top of all nichoid blocks are shown in Figure 10B, where 0.4 dyne/cm^2^ was intentionally set as maximum scale. For all blocks we have calculated a maximum value slightly lower than 1 dyne/cm^2^ (0.947).

### WSS Distribution on the Bottom of Perfusion Chamber

**FIGURE 11:**
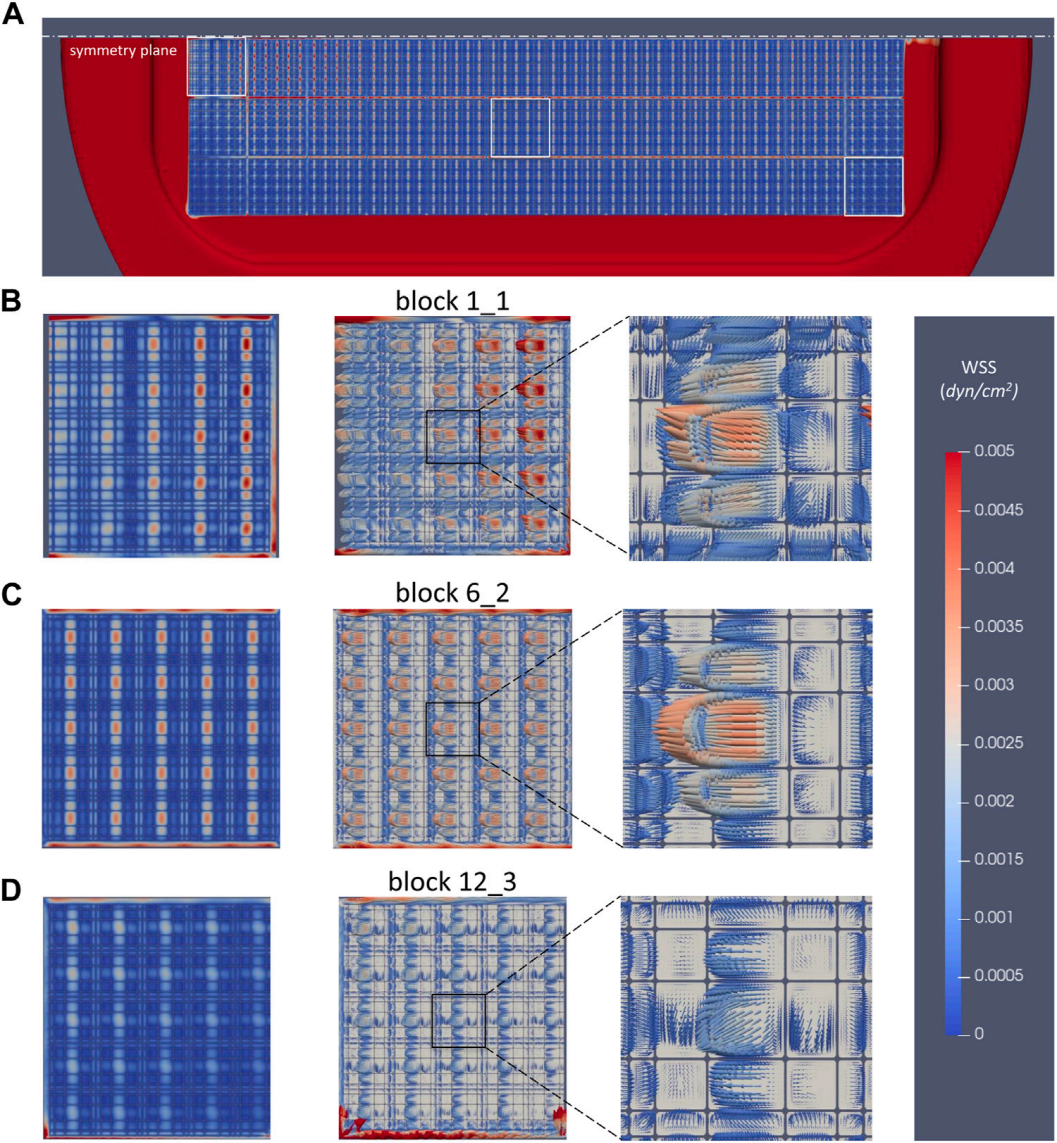
Wall shear stress distribution on the bottom of perfusion chamber. **(A)** WSS under all nichoid blocks, view from top; **(B)** WSS pattern and vectors under the nichoid block 1_1; **(C)** WSS pattern and vectors under the nichoid block 6_2; **(D)** WSS pattern and vectors under the nichoid block 12_3.

The microflow environment inside each 3D niche give rise of micro-shear stresses on the bottom side of the perfusion chamber. These are presented in Figure 11. The WSS distribution under all nichoid blocks is very similar with the velocity magnitude pattern (Figure 11A). Note however, the maximum scale which was set to 0.005 dyne/cm^2^.

In the same manner, patterns and WSS vectors were depicted in Figures 11B–D in zones under the nichoid blocks 1_1, 6_2, and 12_3. Interesting enough, in the smaller niches the WSS vectors are oriented as the mainstream flow, whereas in the bigger niches, the WSS vectors are oriented in opposite direction due to the micro-recirculation flows.

## Discussion

In this study we characterized the microflow inside an array of 3D nichoid blocks manufactured on the bottom of the perfusion chamber of the millifluidic bioreactor commonly known as MOAB-NICHOID. To perform this, we used computational fluid dynamics simulations to compute the flow fields inside a very fine grid resembling the scaffold microenvironment. In particular, the model geometry and boundary conditions corresponded to the macro-environment formed by the perfusion chamber together with the microenvironment created by the 3D scaffold multi-array architecture. We have unveiled a very complex fluid field with myriads of secondary, backward, and local recirculation microflows induced by the intricate architecture of the nichoid scaffold. At the same time, the microflow inside the array of nichoid blocks is fed and influenced by the mainstream flow developed in the perfusion chamber of MOAB-NICHOID device.

In post-processing the results, we have established an objective method to analyze each nichoid block by its position in the matrix array. For the sake of clarity and space here we only presented flow analysis images for three representative blocks i.e., one near the flow entrance, the second in the middle and the third near the flow exit of the perfusion chamber.

The biggest challenge in our study was to generate the computational grid for all nichoid blocks. On the one hand the bigger the number of refined cells, the better will be characterized the flow inside the 3D nichoid scaffold. On the other hand, such a big mesh requires proportionally high computational resources for the numerical solving of the solution, as well as similar resources for post-processing. We therefore resolved this issue by relying on a cloud HPC service that allows scaling of the computational power on-demand. Although we meshed only half of the geometry by taking advantage of the symmetry of the system, the final mesh had more than 188 million cells. It is worth noting that such a huge number of cells is not common in the biomedical literature, where computational grids up to 10 million cells are considered big and are usually used in patient-specific transitional or turbulent flow studies (Hose et al., 2019).

The velocity map of the mainstream flow reveals the highest velocity ( ∼ 7 cm/s) in the two vertical cylinders for inlet and outlet and much lower velocities ( <0.5 cm/s) in the main body of the perfusion chamber. There is a slight acceleration of the fluid in both zones of conjunction between the two vertical cylinders and the main body and a recirculation zone, involving also the nichoids, forms under the flow entrance but not under the exit flow area (Figure 7). For the perfusion chamber hosting the array of 3D nichoids we have calculated a pressure drop of 0.19 mmHg corresponding to the inflow volumetric flow rate of 0.45 ml/min. This is in line with the pressure drop over the flow chamber calculated by considering the whole device flow circuit (Table 3). Thus, we were able to draw the characteristic flow-pressure curves for the MOAB-NICHOID bioreactor for either water or medium for cells. Considering that these flow-pressure relationships are mostly linear (Supplementary Figure S4), our results may help researchers in setting future experiments with this device under flow conditions.

The flow inside the 3D scaffold for cell culture is extremely complex but is at very low velocities. For instance, we have calculated an in-plane maximum velocity of 76 μm/s at 28.6 μm, decreasing up to 2.1 μm/s at 8.6 μm from the bottom of perfusion chamber.

The WSS was relatively high (approx. 1 dyne/cm^2^) on the top rods of nichoids which are hit directly by the mainstream flow, whereas it was three orders of magnitude lower on the bottom of perfusion chamber under the nichoid blocks. Interestingly, the flow inside every single niche resembles the flow in the classical lid-driven cavity problem where the fluid in a cubic or rectangular cavity is moved by the tangential in-plane motion of the top bounding wall (Shankar and Deshpande, 2000). In fact, as seen in Figure 9B a primary vortex develops in the center of the niche. Development of such recirculation zones has direct implication in the development of WSS on the bottom of niche, that will be directed opposite the main flow at the top of niche. However, it is worth noting that such microenvironment may only exist *in vitro* experiments when the scaffold is free of cells, or for some limited time at the beginning of cell seeding process. At this stage of the research, we know that cell seeded for 2 weeks in 3D non-perfused nichoids grow and proliferate, modifying profoundly the geometry of scaffold void, with a consequent reduction in the free volume (Remuzzi et al., 2020). Here we can only speculate that the cultured cells positioned on top of the scaffold will sense a similar shear stress found here (i.e., in the order of 1 dyne/cm^2^, Figure 10A). The hydrodynamic stress exerted by the flow on cultured cells inside the 3D nichoids may still be estimated by fine reconstruction of the porous-like mesh by micro-CT (Cioffi et al., 2006; Raimondi et al., 2006) or nano-CT (Vojtova et al., 2019) images.

Regarding the adequacy of our device for stem cell cultures, we predicted fluid velocities varying between 2 μm/s near the bottom of the nichoid, up to 80 μm/s near the top surface of the nichoid, lapped by the culture medium flow. These velocity gradients generated fluid shear stresses varying between 0.001 dyne/cm^2^ at the bottom of the nichoid, up to 1 dyne/cm^2^ near the top of the nichoid grid. The lower values predicted near the bottom of the nichoid correspond well with physiologically-relevant levels of interstitial fluid velocity (of average 0.6 μm/s, up to 2 μm/s) measured *in situ* in tissue (Chary and Jain, 1989). This implies that the MOAB-NICHOID allows to reproduce *in vitro* a realistic hydrodynamic 3D microenvironment surrounding adherent cells, for example mimicking the interstitial flows sensed by mesenchymal or hematopoietic progenitors resident in the perivascular interstitium. A correct reproduction of interstitial flow is in fact crucial to engineering *in vitro* aspects of tissue morphogenesis and vascular sprouting, which are known to depend on flow velocity and niche topology (Rutkowski and Swartz, 2007; Song and Munn, 2011). In this regard, the presence of cells proliferating in the nichoid grid and the consequent reduction in free volume should slightly decrease the fluid velocity to values below 2 μm/s, which would still fall well within physiologically-relevant levels of interstitial fluid velocity.

Our present study approaches future developments like analysis of microflow when cells are cultured in the MOAB-NICHOID bioreactor, where the fluid shear stress exerted on cells affects cell response, or even simulation of macromolecules and/or nanoparticles transport inside the device to help new drug discovery processes.

## Conclusion

In conclusion, in the present study we have characterized the microenvironment of 3D scaffold for stem cells that was manufactured on the bottom of the perfusion chamber of the MOAB-NICHOID bioreactor. The flow microenvironment inside the 3D nichoids is very complex, with myriads of secondary, backward, and local recirculation microflows induced by the intricate architecture of the nichoid scaffold. Characterization of the spatial fluid flow conditions inside the 3D scaffold is essential to understand the convective transport of solute, the fluid-induced cells behavior, and to control tissue development.

## Data Availability

The original contributions presented in the study are included in the article/Supplementary Material, further inquiries can be directed to the corresponding authors.
