# Supplementary material for: Characterization of the Microflow Through 3D Synthetic Niche Microenvironments Hosted in a Millifluidic Bioreactor
Source: Front Bioeng Biotechnol. 2021 Dec 17;9:799594. doi: 10.3389/fbioe.2021.799594 (PMC8718690; doi:10.3389/fbioe.2021.799594)
Supplement: Supplementary file 1 [file Presentation1.pdf]

Supplementary Material

Characterization of the Microflow Through 3D Synthetic Niche Microenvironments Hosted in a Millifluidic Bioreactor

Bogdan Ene-Iordache, Chiara Emma Campiglio, Manuela Teresa Raimondi and Andrea Remuzzi

Table of Contents

1 Validation of the pressure losses in the MOAB flow system.....2

1.1 Experimental measurement of the pressure inside the MOAB circuit.....2

1.2 Generation of MOAB flow circuit mesh.....2

1.3 CFD simulation of the MOAB flow system.....3

1.4 Numerical simulation results.....4

1.4.1 The pressure drop over the MOAB flow circuit chamber.....4

2 Meshing study for one nichoid block.....6

2.1 Introduction.....6

2.2 Grid generation testing.....6

2.3 Best mesh for one nichoid block.....7

3 References.....9

1 Validation of the pressure losses in the MOAB flow system

1.1 Experimental measurement of the pressure inside the MOAB circuit

We measured experimentally the pressured drop over the MOAB flow system. As shown in Figure 4 in the main text, the experimental flow system is composed of a syringe pump (Pump 11, Harvard Apparatus, Holliston, MA) that circulates water at 24°C in a silicone rubber tubing circuit in which a pressure transducer (MP150 Biopac Systems, Goleta, CA) and the MOAB device are included. For a flow rate of 2.295 mL/min, the experimentally measured pressure drop was 11.86 mmHg.

1.2 Generation of MOAB flow circuit mesh

A detailed model of the MOAB flow circuit was generated using a CAD program (FreeCAD, https://www.freecadweb.org/). As show in Figure 4B in the main text, starting from right to left, this model consists of the inlet luer, of diameter 2.6 mm and length 30 mm, then a small part of length 1 mm and 4mm diameter, from which the microbore tubing of length 33 mm and diameter 0.5 mm starts. Then the fluid enters the scaffold chamber, and after the chamber the fluid is exiting the system through the outflow microbore tube that ends with a luer of length 10 and diameter 4 mm. The entire fluid path surface was exported in stereolithography (STL) file format and then was sub-divided into separate parts representing the patches used in CFD simulations like inlet, outlet, and walls, as shown in Supplemental Figure 1.

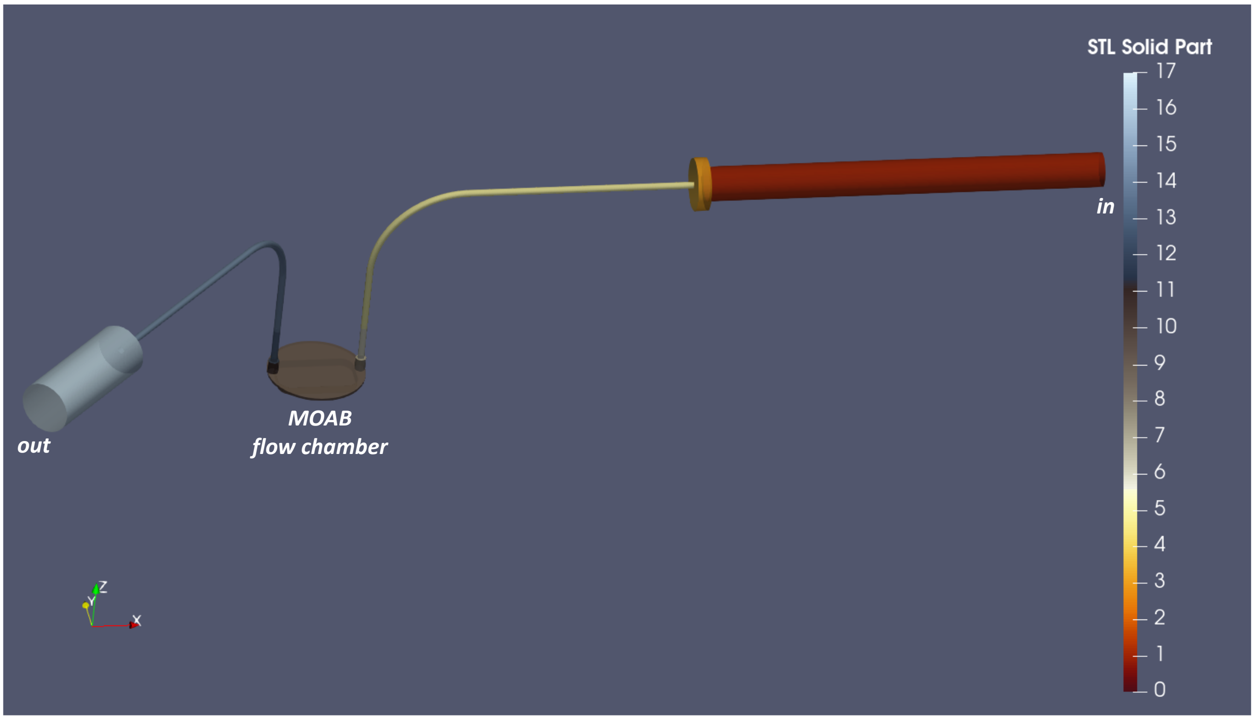

Supplemental Figure 1. STL model of the MOAB flow system. The fluid path was divided into 18 parts that will be individual entities for the boundary conditions of CFD analysis.

The computational grid of the MOAB flow circuit was generated with the *snappyHexMesh* pre-processor of the OpenFOAM CFD toolbox (OpenCFD Ltd, 2020). A fine mesh composed of more than 5.7 million cells was generated, as detailed in Supplemental Figure 2.

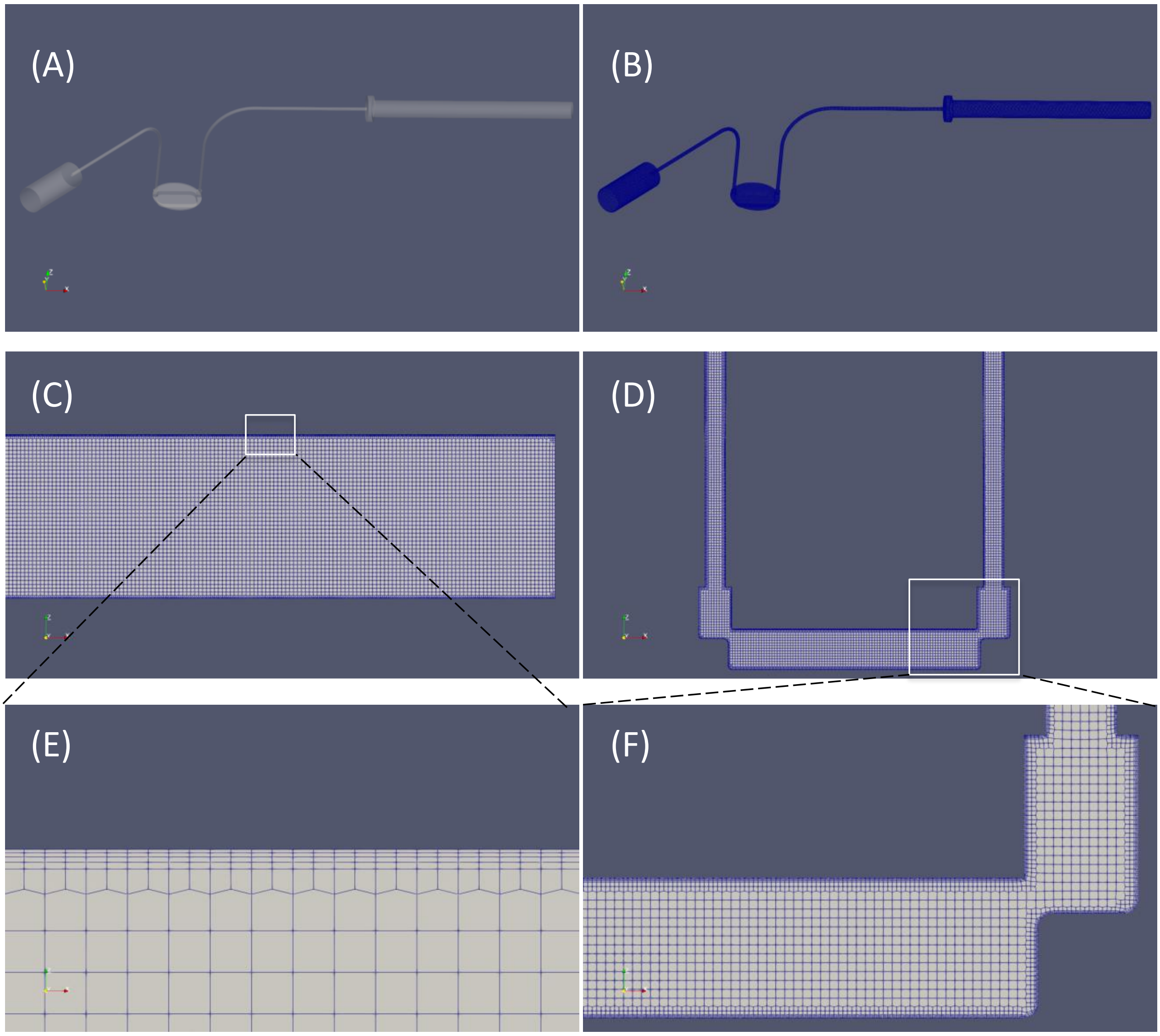

Supplemental Figure 2. Computational mesh of the MOAB circuit. (**A**) Isometric view of the circuit external surfaces; (**B**) Isometric view of the fine numerical grid; (**C**) Computational mesh near the entrance luer; (**D**) View of a section cutting the scaffold chamber; (**E**) View of (D), detail of the grid near the wall showing boundary layers; (**F**) View of (D), detail of the grid near the wall showing inner cells and the boundary layers.

1.3 CFD simulation of the MOAB flow system

Steady simulations were run using the solver *simpleFoam* solver of OpenFOAM suite (OpenCFD Ltd, 2020). The fluid considered was the same used in experimental measurements of pressure, i.e., was water at 24°C, for which we assumed density (ρ) equal to 0.9973 $g{\cdot}cm^{-3}$ and dynamic viscosity (μ) equal to 0.0091 $g{\cdot}cm^{-1}{\cdot}s^{-1}$. Newtonian rheology model was assumed. As boundary conditions, we set a constant volumetric flow rate at the inlet equal to that of the infusion pump used in the experimental setting, which was 2.295 mL/min. On the outlet a *zero-pressure* condition was set, and *no-slip* condition was set on all system walls.

1.4 Numerical simulation results

The numerical simulations resolve for the pressure and velocity fields within the MOAB flow system circuit.

1.4.1 The pressure drop over the MOAB flow circuit chamber

The pressure drop over the MOAB circuit is shown in Supplemental Figure 3.

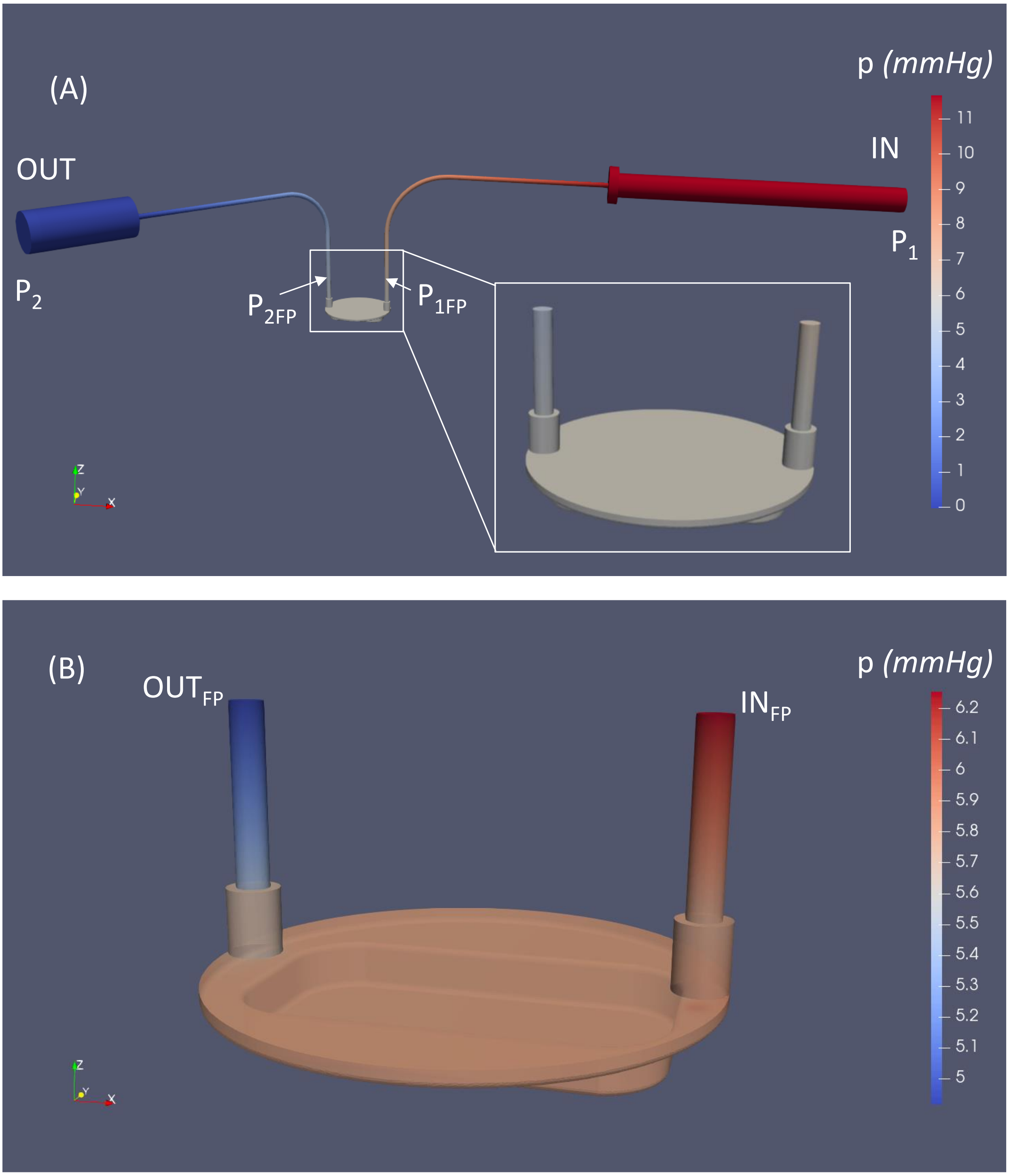

Supplemental Figure 3. Pressure in the MOAB circuit. (**A**) The pressure drop over the MOAB circuit; (**B**) The pressure drop over the flow chamber of the scaffold.

As shown in Supplemental Figure 3, the highest-pressure gradients take place in the inflow and outflow microbore tubes, where the diameter is minimum (0.5 mm). For the flow rate used in the experimental setting (e.g., 2.295 mL/min, water at 24°C), the pressure drop (calculated as P_1 - P_2 as shown in Supplemental Figure 3A) was 11.67 mmHg, while the pressure drop only in the flow chamber (calculated as P_{1FP} - P_{2FP} as shown in Supplemental Figure 3A) was 1.34 mmHg. Since the experimentally measured pressure drop was 11.86 mmHg, we concluded that the numerical simulations may well predict the pressure losses over the MOAB flow system.

To derive the characteristic flow-pressure curve of the whole circuit, as well as of the scaffold chamber alone, we have run steady simulations with a set of volumetric flow rates and medium commonly used in *in vitro* experiments on cells using the MOAB device. For the medium for cells at 37°C we assumed density (ρ) is 0.99 $g{\cdot}cm^{-3}$ and dynamic viscosity (μ) is 0.0076 $g{\cdot}cm^{-1}{\cdot}s^{-1}$ (Franzoni et al., 2016). We therefore derived characteristic pressure vs. flow rate curves of the MOAB system as well as of the scaffold chamber for both fluids, as shown in Supplemental Figure 4.

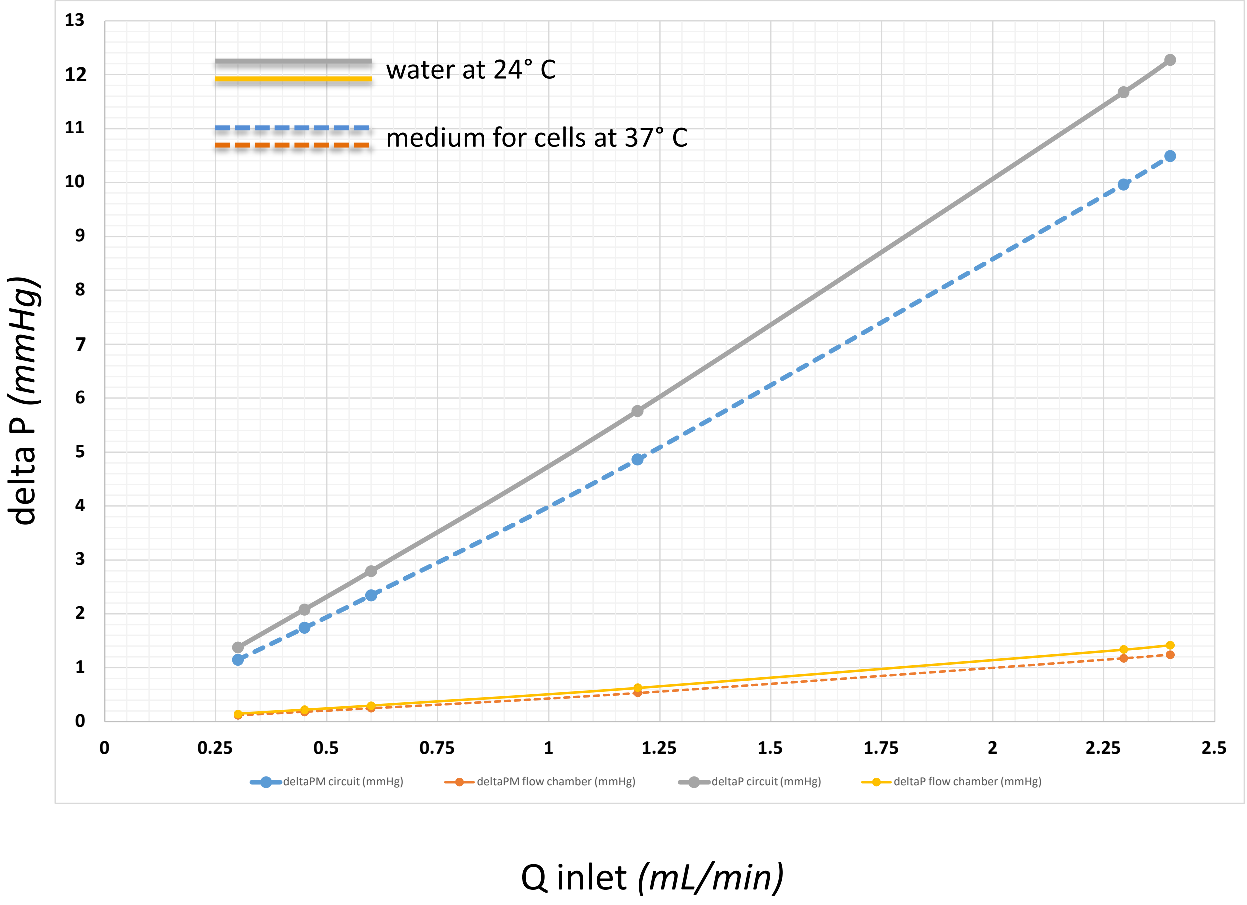

Supplemental Figure 4. Pressure drops over the MOAB circuit and perfusion chamber as function of inlet volumetric flow rate, for two fluids (water at 24 °C and medium for cells at 37 °C).

2 Meshing study for one nichoid block

2.1 Introduction

The CAD surface (in STL format) of a basic nichoid block is shown in Figure 3 in the main text. The entire block is 450x450 μm wide and 33 μm height. We decided to perform a preliminary meshing study for one basic nichoid block before proceeding to the final grid generation.

2.2 Grid generation testing

Initially, the STL file was rescaled such as to match 434 μm width as we found in the dimensional analysis described in the main text. Then, a parallelepiped of dimensions 454x454x40 (WxLxH) μm was chosen as overall 3D domain, in such a way as to contain the nichoid block on the bottom and aligned in the middle. For mesh generation we tested both *snappyHexMesh* and *cfMesh* pre-processors of OpenFOAM v2012 suite.

Some attempts were made with *snappyHexMesh* to generate accurate grids of the scaffold block, which revealed that meshing the nichoid is a challenging task. Several meshes of 2.4 and 2.6 million cells were generated, for which, even though at macroscopic examination the mesh seemed good, when observed in detail, it had distorted or even missing cells, as exemplified in Supplemental Figure 5.

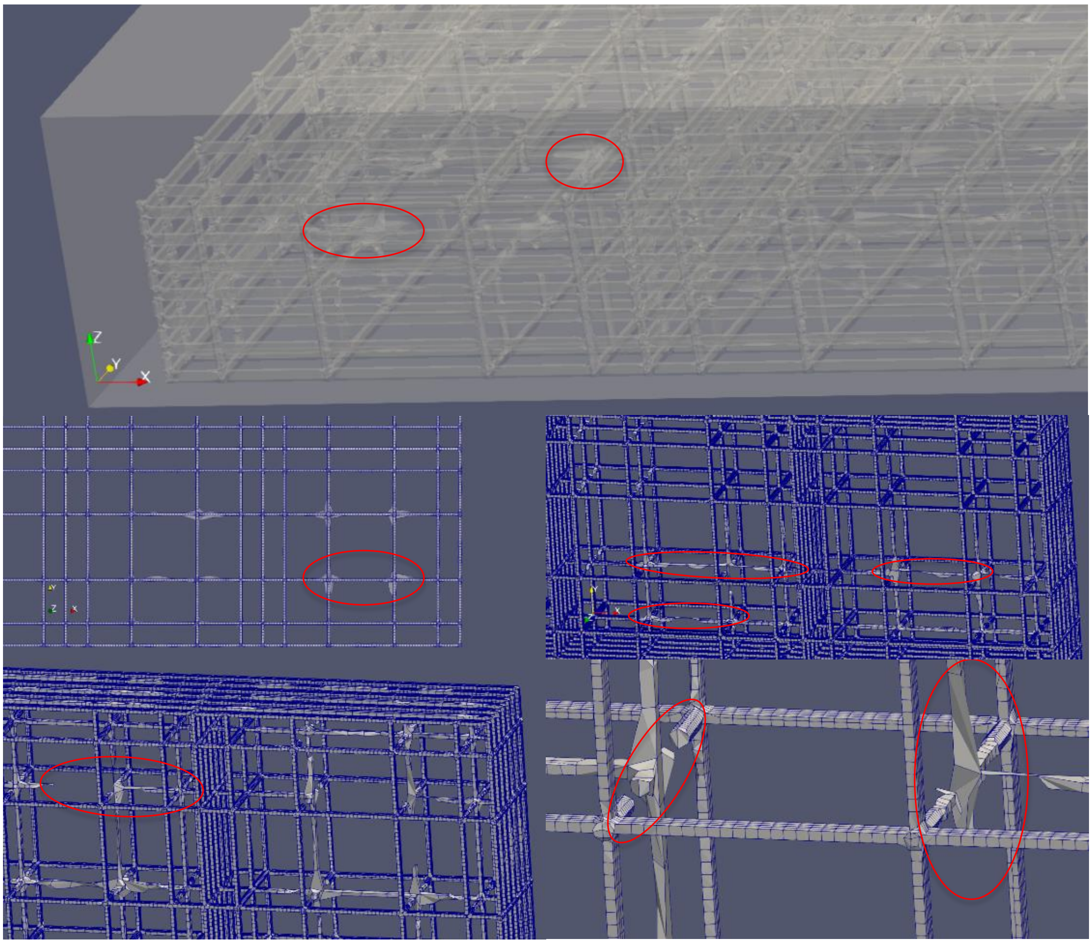

Supplemental Figure 5. Computational grid generated with *snappyHexMesh*. View of mesh surface and details showing common meshing errors at rods intersection.

The grid errors shown in Supplemental Figure 5, like distorted or missing cells for vertical rods, indicate that the mesh must be further refined to obtain a numerical grid as close as possible to the geometry of nichoid structure. We performed further refinements by increasing the number of cells, especially near the rods since the obtainment of the best mesh with *snappyHexMesh* composed of more than 5.5 million cells.

Similarly, several tries were made with *cfMesh* to generate the computational grid for one nichoid block, until the correct parameters were set. A final, best mesh composed of more than 5.4 million cells was thus obtained with *cfMesh* pre-processor. The quality of best meshes obtained with these two different mesh generators was assessed by means of *checkMesh* utility (OpenCFD Ltd, 2020) and the output is presented in Supplemental Table 1.

Supplemental Table 1. Characteristics of best mesh of one nichoid block obtained with two mesh generators.

mesh generator	***snappyHexMesh***		***cfMesh***	
Mesh stats				
points:	7574190		7082916	
faces:	18812771		18010684	
internal faces:	17060762		16268303	
cells:	5591155		**5428695**	
faces per cell:	6.41612		**6.31441**	
boundary patches:	7		7	
Overall number of cells of each type:				
hexahedra:	4697438	84.0%	**4782083**	**88.1%**
prisms:	34269	0.6%	8116	0.1%
wedges:	0		0	
pyramids:	0		17883	
tet wedges:	0		0	
tetrahedra:	0		8164	
polyhedra:	859448	15.4%	**612449**	**11.3%**
Checking geometry...				
	Max aspect ratio = 6.15523 OK.		**Max aspect ratio = 5.13409 OK.**	
	Mesh non-orthogonality Max: 63.7446		Mesh non-orthogonality Max: **53.4485**	
	Non-orthogonality check OK.		Non-orthogonality check OK.	
	***Error in face pyramids: 1 faces are incorrectly oriented.		Face pyramids **OK**.	
	***Max skewness = 16.5303, 33 highly skew faces detected		Max skewness = 1.87424 **OK**.	
	Failed 2 mesh checks.		Mesh **OK**.	

[1] In bold type the characteristics considered better.

2.3 Best mesh for one nichoid block

As may be observed in Supplemental Table 1, the two computational grids have 5591155 and 5428695 cells, for the *snappyHexMesh* and *cfMesh*, respectively. In terms of cells shape, the numerical grid is composed of 4698515 (84%) and 4782083 hexahedra (88.1%), respectively. Regarding quality tests that may entail the numerical solution, the *snappyHexMesh* failed two mesh checks, whereas *cfMesh* passed all quality checks OK. For these reasons, and because the

mesh has less cells, we decided to keep as best mesh that generated with *cfMesh*, which is shown in Supplemental Figure 6.

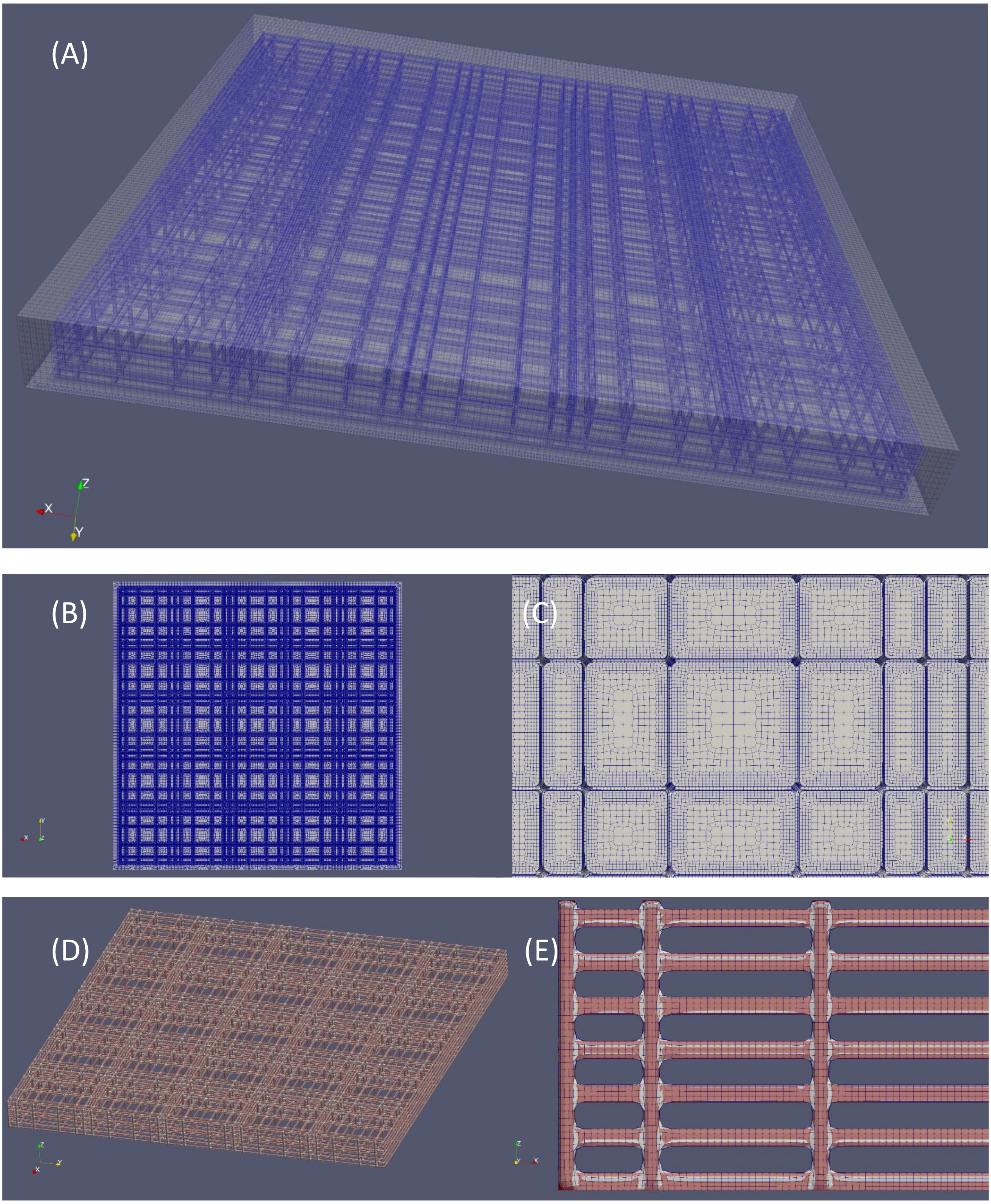

Supplemental Figure 6. Best mesh of a basic nichoid block. (**A**) Isometric view with transparency; (**B**) Bottom view; (**C**) Zoomed view of (B) showing detail of mesh refinement; (**D**) Mesh walls superimposed with the original STL surface (pink); (**E**) Zoomed view showing differences between the final mesh (grey and dark blue edges) and the input STL file (pink).

3 References

Franzoni, M., Cattaneo, I., Longaretti, L., Figliuzzi, M., Ene-Iordache, B., and Remuzzi, A. (2016). Endothelial cell activation by hemodynamic shear stress derived from arteriovenous fistula for hemodialysis access. *Am J Physiol Heart Circ Physiol* 310**,** H49-59.

OpenCFD Ltd. About OpenFOAM® [Online]. Available: https://www.openfoam.com/ [Accessed November 2020].
